# Improved Survival From Graft-versus-host Disease Following Pediatric Small Intestinal Transplantation Through Reduction in Systemic Immunosuppression Altering T-cell Chimerism Dynamics

**DOI:** 10.1097/TXD.0000000000001830

**Published:** 2025-06-27

**Authors:** Sandeep Potluri, Sarah Lawson, Shyla Kishore, Malobi Ogboli, Jane Hartley, Arun Alfred, Yvonne Wilson, Darius F. Mirza, Khalid Sharif, Girish Gupte

**Affiliations:** 1 Birmingham Women’s and Children’s NHS Foundation Trust, Birmingham, United Kingdom.; 2 Cancer and Genomic Sciences, University of Birmingham, Birmingham, United Kingdom.; 3 Department of Haematology, The Rotherham NHS Foundation Trust, Rotherham, United Kingdom.; 4 Immunology and Immunotherapy, University of Birmingham, Birmingham, United Kingdom.

## Abstract

**Background.:**

Graft-versus-host-disease (GvHD) is an infrequent but serious complication of small intestinal transplantation in children, which is associated with a very poor prognosis. This study evaluated a novel strategy of managing GvHD in these patients through a reduction in immunosuppression.

**Methods.:**

We conducted a retrospective review 108 consecutive pediatric patients at our center between 2005 and 2021, who had small intestinal transplantation. We assessed clinical features and outcomes as well as laboratory chimerism studies in cohorts of patients before and following a change in treatment strategy for GvHD from intensification to reduction in immunosuppression.

**Results.:**

Fourteen percent of pediatric patients developed GvHD after small intestinal transplantation. A change in treatment strategy to a reduction in immunosuppression led to significantly improved overall survival (log rank *P* = 0.015). This improved survival correlated biologically with altered T-cell chimerism dynamics in blood; in patients who had a reduction in immunosuppression, there was abrogation of the rise in donor T-cell chimerism over time seen in the blood of patients who instead had intensification of their immunosuppression. This may be because of permitting recipient lymphocytes to have a host-versus-graft effect and outcompete donor-derived lymphocytes.

**Conclusions.:**

Our results demonstrate that that altering the immunosuppressive therapy strategy, following clinical manifestations of GvHD such as a typical skin rash, from intensification to a reduction in immunosuppression led to significantly improved survival.

## Graphical abstract

**Figure:**
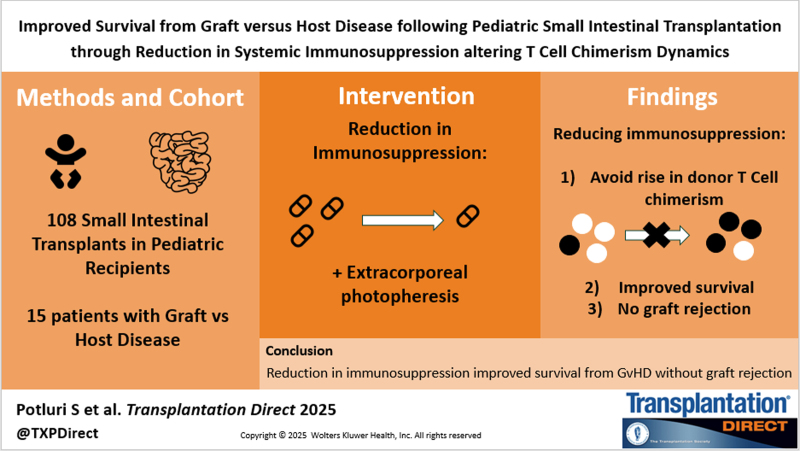

Survival from pediatric intestinal transplantation has been improving over the last 3 decades^1^ since the first case reports of long-term survivors were described.^2,3^ Graft-versus-host disease (GvHD) is a serious systemic complication after intestinal transplants, which has been described with an incidence of 5%–10%^4-6^ and remains a significant contributor to mortality in these patients. This incidence of GvHD in the small intestinal transplantation context is higher than the 1% reported after liver transplantation^7^ but lower than the 40%–50% reported after bone marrow transplant.^8^

The clinical picture of GvHD is variable as different organs that may be involved. The skin is the commonest organ affected by GvHD and has been reported in up to 8%–10% of pediatric intestinal transplant recipients. Other organs can be affected too, including the native gastrointestinal tract, native liver, lungs, bone marrow, and eyes.

Treatment of acute skin GvHD postintestinal transplantation has historically involved intensification of immunosuppression, following on from the paradigm established in the bone marrow transplantation context.^9^ However, outcomes have been unsatisfactory with mortality of >50% in some case series.^4^ Mortality and morbidity related to GvHD posttransplant not only relate to the disease itself but also because of the detrimental effect of aggressive immunosuppression used to stop donor alloreactive cells causing GvHD, which may be relatively resistant to immune suppression.

Here, we share our experience over the past 2 decades of changing our immunosuppression strategy to improve outcomes.

## MATERIALS AND METHODS

We performed a retrospective review of case notes and electronic database, of 108 consecutive small intestinal transplant recipients between 2005 and 2021 at Birmingham Children’s Hospital, United Kingdom.

Skin GvHD was staged using methods established in the bone marrow transplantation context, depending on the percentage of body surface area affected by rash and whether bullous formation or desquamation occurred.^10^ Skin GvHD was graded based on histological findings.^6^

Between 2004 and 2015 (cohort 1), patients with GvHD post small intestinal transplant were treated with an overarching strategy of increasing immunosuppression, as described by other centers.^4,11^

First line treatment for patients included all of:

- Increase in tacrolimus dose to achieve trough tacrolimus levels of 7–15 ng/mL or sirolimus to achieve trough levels of 5–8 ng/mL in patients intolerant of tacrolimus.
- Intravenous methylprednisolone pulses at 10 mg/kg (capped at 400 mg).
- Infliximab (10 mg/kg), daclizumab (1 mg/kg), or basiliximab (dose dependent on weight of recipient; approximately 1 mg/kg capped at 20 mg given on day of transplant and 4 d after).
- Mycophenolate mofetil at 10 mg/kg/dose twice daily.

If there was no improvement, second-line therapies included:

- Mesenchymal stromal cells.
- Alemtuzumab (0.3 mg/kg once weekly for 2 doses) or antithymocyte globulin (1.5 mg/kg for 4 or 5 doses).
- Extracorporeal photopheresis twice weekly.

After 2015 (cohort 2), patients were treated with a novel protocol with a strategy of generally decreasing immunosuppression, whilst giving just enough to prevent graft rejection.

For patients with just stage I skin GvHD, the dose of tacrolimus was reduced to achieve trough levels of 2–4 ng/mL, and all other immunosuppression was stopped.

For patients with stage II-III skin GvHD or signs of other organ GvHD, tacrolimus was reduced and other immunosuppression stopped as above but additionally a single dose of 10 mg/kg intravenous methylprednisolone (capped at 400 mg) was given together with extracorporeal photopheresis (ECP) and mesenchymal stromal cells.

For patients with stage IV skin GvHD, as well as the above treatment for stage II-III skin GvHD, all immunosuppression including tacrolimus was stopped.

Immunosuppression levels were monitored in all patients daily in the weeks immediately after transplant. Monitoring was gradually spaced out to 3 times a week and then weekly and then fortnightly, over time and as the patients has levels more consistently within the desired therapeutic ranges.

Monitoring for rejection and intestinal GvHD was through surveillance endoscopies and colonoscopies with biopsies taken of the graft as well as of native bowel. For patients who had concomitant liver transplants, surveillance ultrasound scans of the liver graft were performed with quantification of venous and arterial flow as well as of the hepatic artery resistive index. The frequency of these procedures was clinically guided and dependent on findings in previous studies.

Peripheral blood chimerism studies were carried out at the West Midlands Regional Genetic Laboratory. Peripheral blood was separated into a CD3^+^ T-cell fraction by magnetic-activated cell sorting. In the case of sex-mismatched transplants, chimerism was analyzed by interphase fluorescence in situ hybridization (FISH) studies using FISH probes specific to the X chromosome centromere or specific heterochromatic region in the long arm of the Y chromosome. In the case of sex-matched transplants, chimerism was analyzed by assessing DNA microsatellite/short tandem repeat markers.

## RESULTS

Fifteen of 108 small bowel transplant recipients developed skin GvHD between 2005 and 2021. Table 1 shows the patient demographics, indication for solid organ transplantation, type of intestine inclusive transplantation, and stage of GvHD.

**TABLE 1.** Patient demographics

Patient No.	Cohort	Underlying diagnosis	Age at transplant	Indication for transplant	Type of intestinal graft	Year GvHD diagnosed	GvHD Stage
1	1	Gastroschisis	5 y 5 mo	Irreversible IF	Small bowel and pancreas	2004	4
2	1	Gastroschisis	1 y 1 mo	Recurrent CVL sepsis and Progressive IFALD	Liver, pancreas, and small bowel	2007	1
3	1	Gastroschisis	1 y 6 mo	Irreversible IF and Recurrent CVL sepsis	Liver, pancreas, and small bowel	2009	4
4	1	Necrotizing enterocolitis	2 y	IF and IFALD	Liver, pancreas, and small bowel	2012	4
5	1	Necrotizing enterocolitis	10 mo	Irreversible IF and recurrent CVL sepsis	Liver, pancreas, and small bowel	2014	2
6	1	Microvillus inclusion disease	5 y 3 mo	Irreversible IF	Small bowel and pancreas	2012	3
7	1	Microvillus inclusion disease	3 y 4 mo	IF and IFALD	Small bowel and pancreas	2012	3
8	1	Pseudo obstruction Intestinal dysmotile syndrome	9 y 2 mo	Recurrent CVL sepsis and progressive IFALD	Multivisceral	2006	1
9	1	Megacystis microcolon-intestinal hypoperistalsis syndrome (MMIH)	1 y 9 mo	Irreversible IF and recurrent CVL sepsis	Modified multivisceral	2010	4
10	1	Megacystis-Megaureter intestinal hypoperistalsis syndrome	1 y 2 mo	IF and IFALD	Multivisceral	2014	3
11	2	Malrotation with mid-gut volvulus	2 y 10 mo	IF and IFALD	Liver, pancreas, and small bowel	2015	2
12	2	Pseudo obstruction intestinal dysmotile syndrome	10 y 3 mo	Irreversible IF and recurrent CVL sepsis	Liver, pancreas, and small bowel	2015	3
13	2	Gastroschisis/SBS	1 y 3 mo	IF and IFALD	Liver, pancreas, and small bowel	2021	4
14	2	Hepatoblastoma	7 y 10 mo	Hepatoblastoma with IVC involvement	Multivisceral with splenectomy	2020	4
15	2	Gastroschisis/short bowel syndrome secondary to volvulus/stoma in situ	10 y 2 mo	IF and IFALD. Bleeding varices and PN dependent	Liver, pancreas, and small bowel	2020	2

CVL, central venous line; IF, intestinal failure; IFALD, intestinal failure-associated liver disease; IVC, inferior vena cava.

In most patients, reduction of small bowel, liver or both were necessary for size mismatch reasons. All patients had organs from donation after brainstem death donors ranging from 6 mo to 17 y of age. Warm ischemia time ranged between 20 and 65 min and cold ischemia time ranged between 235 and 545 min.

Skin was the primary organ affected with GvHD, and all 15 patients had skin GvHD. Eight patients had other organs also affected by GvHD including 2 patients with native intestinal involvement, 5 patients with ocular involvement (corneal ulceration or lens perforation), and 2 patients with native liver involvement (with deranged liver function and cholestasis).

The median time for diagnosis of skin GvHD from the date of transplantation was 3 mo with a range of 3 wk to 12 mo. The typical clinical pattern of skin GvHD observed was an erythematous and itchy rash on palms and soles that later spread to include the trunk within 2–4 w k.

Two patients had stage 1 GvHD, 4 patients had stage 2, 4 patients had stage 3, and 5 patients had stage 4 GvHD (Table 1). In 12 of these patients, a clear histological diagnosis of skin GvHD could be made and a histological grade assigned; 8 patients had Grade 1–2 skin GvHD, 3 had grade 3 GvHD, and 1 had grade 4 GvHD. Similar to findings by others, we found a poor correlation between GvHD stage and grade.^12^

All patients were discussed in multidisciplinary team meetings comprising hepatologists, transplant surgeons, dermatologists, and hematologists. All patients received topical emollients, which provided symptomatic relief but little or no clinical improvement of the skin lesions. Eleven of 15 patients received intravenous steroid therapy at diagnosis, and 1 patient was given oral prednisolone. The majority responded well initially but most developed either steroid-dependent or refractory skin GVHD.

In patients treated before 2015 (cohort 1), immunosuppression was intensified as described previously. A variety of immune modulators were used as part of first-line treatment; between 2005 and 2008, 4 patients had infliximab (tumor necrosis factor alpha inhibitor) or daclizumab (interleukin [IL]-2 receptor antagonist). Between 2010 and 2012, 4 patients received infliximab and basiliximab (IL-2 receptor antagonist). Two patients went on to receive alemtuzumab (anti-CD52 antibody) as second-line therapy. Although a variety of immune modulators were used in that cohort, there were invariably only minimal clinical improvements.

Between 2010 and 2014, in addition to immunomodulatory agents, 5 patients were treated with bone marrow–derived mesenchymal stromal cells, and some clinical response was seen in 4 patients. Between 2012 and 2014, 3 patients also received twice weekly ECP and 2 of them had some clinical improvement in skin GvHD.

All 10 patients in cohort 1 died because of infection-related complications or progressive GvHD. Six patients had some modest improvements in their skin GvHD but then died because of infections including *Staphylococcus aureus* sepsis, Pseudomonal infections, fungal brain abscess, and encephalitis.

Recognizing the relatively poor clinical outcomes and high rates of severe infections, from 2015 (cohort 2), we altered our strategy of treatment to permanently reduce immunosuppression. In this second cohort of patients, 2 patients with stage 2 skin GvHD had tacrolimus doses reduced to achieve lower trough levels of 2–4 ng/mL and all other immunosuppression stopped. For 3 further patients with stage 3 or 4 GvHD, in addition to reduction of tacrolimus doses, they were also treated with twice weekly extracorporeal photopheresis and mesenchymal stromal cells. Four of these patients have had complete resolution of their skin GvHD and are alive without any signs of recurrence of GvHD or of rejection. One patient had progressive GvHD despite these interventions. None of the patients who had reduction of immunosuppression developed graft rejection with a median follow-up of 1341 d . Other than the change in immunosuppression strategy, there were no other significant differences in clinical management or in baseline clinical features of the 2 cohorts of patients.

**FIGURE 1.**
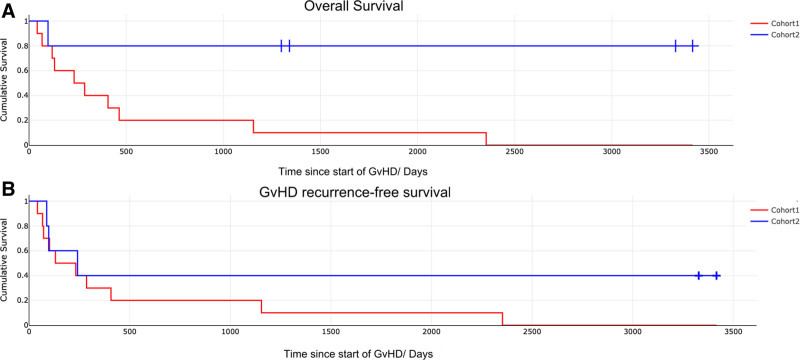
Overall and GvHD-free survival comparing intensification with reduction of immunosuppression. A, Kaplan–Meier plot comparing survival (in days) since establishment of GvHD in cohort 1 where immunosuppression was intensified and cohort 2 where immunosuppression was reduced. Log rank *P* = 0.015. B, Kaplan-Meier plot comparing GvHD recurrence-free survival (in days) since establishment of GvHD in cohort 1 where immunosuppression was intensified and cohort 2 where immunosuppression was reduced. Log rank *P* = 0.21. GvHD, graft-versus-host disease.

Figure 1A shows a Kaplan–Meier plot comparing survival with the contrasting strategies of intensification of immunosuppression and reduction in immunosuppression. A significant improvement in survival was seen with reduction in immunosuppression (*P* = 0.015 by the log rank test) with 80% of patients surviving long-term until the time of last follow-up, compared with no long-term survivors in the patients treated with intensification of immunosuppression. Two patients in cohort 2 each had one episode with a mild recurrence of skin GvHD that needed treatment with topical steroids. Figure 1B shows a Kaplan–Meier plot comparing GvHD recurrence-free survival between intensification of immunosuppression and reduction in immunosuppression cohorts. No significant difference in GvHD-free survival was seen between the 2 cohorts (*P* = 0.19 by the log rank test). However, long-term, 40% of patients with GvHD recurrence-free survival at the time of last follow-up in patients treated with reduction of immunosuppression compared with no patients with GvHD recurrence-free survival at the time of last follow-up when treated with intensification of immunosuppression.

**FIGURE 2.**
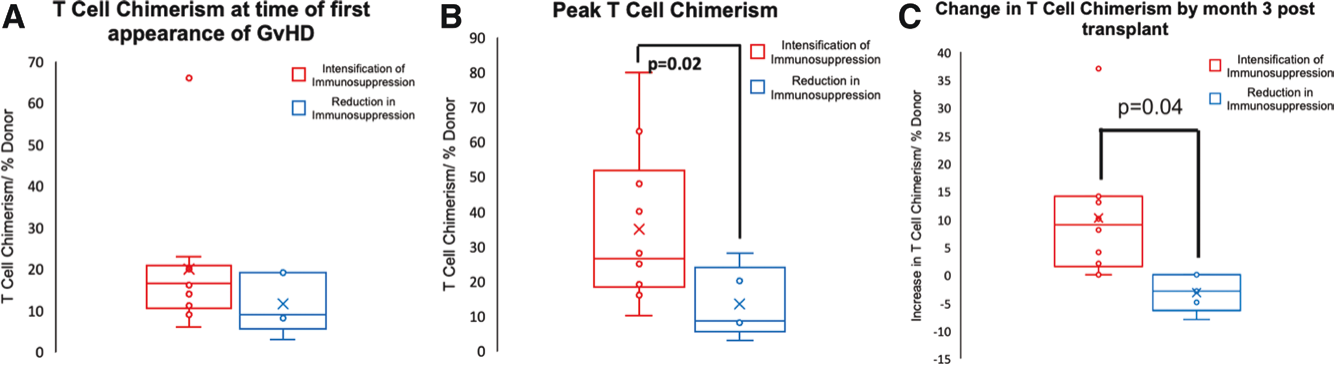
T-cell chimerism dynamics. Box-plots of donor T-cell chimerism at the time of first appearance of GvHD (A) and the peak donor T-cell chimerism (B) and box-plots of the increase in T-cell chimerism by month 3 post onset of GvHD (C). In cohort 1 immunosuppression was intensified, and in cohort 2, immunosuppression was reduced. Statistical analysis was undertaken using a Student *t*-test. GvHD, graft-versus-host disease.

Chimerism studies were undertaken on all patients. Analysis of the donor T-cell chimerism looking for the percentage of donor T cells in peripheral blood showed no difference between 2 cohorts of patients (pre-2005 and post-2005) at first presentation of GvHD (Figure 2A). However, analyzing the maximum T-cell chimerism that patients reached, this showed significantly increased T-cell chimerism in patients who were treated with an intensification of immunosuppression compared with those who had a reduction in immunosuppression (*P* = 0.028) (Figure 2B). This difference was largely because of rising T-cell chimerism when immunosuppression was intensified, which did not happen when immunosuppression was reduced. Crucially, there was a large difference in the change in T-cell chimerism by month 3 post onset of GvHD (*P* = 0.004) (Figure 2C). Patients who were treated with an intensification of immunosuppression generally had an increase in T-cell chimerism by month 3, whereas patients who were treated with a reduction in immunosuppression generally had a decrease in T-cell chimerism. Similarly, when analyzing the change in chimerism by month 3 post onset of GvHD between patients who survived and patients who did not survive, there was a significant difference with generally decreasing T-cell chimerism in survivors and generally increase in chimerism in non survivors (*P* = 0.003).

Within cohort 2, there was little heterogeneity between the types of organs transplanted. However, within cohort 1, 6 patients had concurrent liver transplants but 4 patients did not. The mean time to skin GvHD was 3.4 months in both patients who did and did not have concurrent liver transplants (*t*-test *P* = 0.96). The mean donor T-cell chimerism at time of onset of GvHD was 28% in the patients who did not have concurrent liver transplants and 14.5% in patients who did have concurrent liver transplants (*t*-test *P* = 0.38). The mean peak donor T-cell chimerism was 42.8% in the patients who did not have concurrent liver transplantation and 23% in patients who did have concurrent liver transplants (*t*-test *P* = 0.35). The mean increase in donor T-cell chimerism at 3 mo post onset of GvHD was 17% in the patients who did not have concurrent liver transplantation and 5.7% in patients who did have concurrent liver transplantation (*t*-test *P* = 0.19). Therefore, there were no statistically significant clinical sequelae of having concurrent liver transplantation with small intestinal transplantation.

## DISCUSSION

GvHD is a serious complication of small bowel transplantation and occurred in 15 patients of 108 (14%) over a 16-y period at our center. Mortality was initially high, but after discussion between national experts in intestinal transplantation and stem cell transplantation, a reduction in immunosuppression strategy upon clinical manifestation of GvHD was adopted.

The higher incidence of GvHD in small intestinal transplantation relative to liver transplantation may be in part because of the higher lymphoid burden in intestinal grafts,^13^ and this is particularly the case in pediatric small intestinal grafts that have a proportionately larger lymphoid mass compared with adults.^14^ Although, in our series, most patients had some reduction of the small bowel, liver, or both for size mismatch reasons, the lymphoid mass relative to patient size was likely higher than in most adult patients, which may explain the relatively high rates of GvHD seen compared with series in which adult patients were included.^4-6^

Because of the high rates of acute cellular rejection of grafts of up to 60% described in patients with small intestinal transplants,^15^ immunosuppression strategies are of paramount importance. It has been postulated that in the event of tissue injury, such as from a viral infection, donor lymphocytes are activated; this triggers an immunological response against the recipient, which may manifest as GvHD. However, mortality and morbidity related to GvHD post transplant not only relate to the disease itself but also because of the detrimental effect of aggressive immunosuppression used to stop donor alloreactive T cells causing GvHD, which may be relatively resistant to immunosuppression.

Strategies to tackle GvHD have generally involved steroid intensification and a variety of combinations of anti thymocyte globulin (ATG), sirolimus, ECP, ruxolitinib, IL-2 receptor blockers, and tumor necrosis factor ⍺ antibodies.^4,11^ Other centers performing pediatric small intestinal transplantation have described their experiences and evolution of baseline immunosuppressive strategies. This included the substitution of basiliximab with rabbit ATG by the investigators at Georgetown, United States,^4^ addition of alemtuzumab or rabbit ATG and rituxumab by the investigators at Miami, United States,^5^ addition of daclizumab and rabbit ATG by the investigators at Pittsburgh, United States,^6^ and the addition of rabbit ATG or alemtuzumab to basiliximab by the investigators in Madrid, Spain.^11^

It has also been described that the risk of graft rejection is quite low in patients with GvHD post small intestinal transplantation and a slight reduction in target tacrolimus levels can be achieved without increased rejection.^6^ However, we describe a more radical strategy of reduction in immunosuppression following clinical manifestations of GvHD and did not see any patients develop graft rejection.

We have also demonstrated that the peak donor T-cell chimerism attained by patients with intensification of immunosuppression is significantly greater than those patients in which immunosuppression was reduced, and this may be correlated with the worse clinical outcome. In particular, the change in T-cell chimerism by month 3 post onset of GvHD appeared to be significantly different between the 2 cohorts and this may represent the time window in which interventions in immunosuppression can alter T-cell chimerism.

Indeed, a complex interplay exists between recipient-derived and donor-derived lymphocytes post small intestinal transplant. Donor-derived lymphocytes can engraft and can be found in the peripheral blood of recipients in chimerism studies,^6^ in some cases even after graft enterectomy and in native colonic mucosa.^16^ Similarly, skin biopsy specimens of patients with GvHD also show donor-derived lymphocytes, as confirmed by FISH, Y chromosome polymerase chain reaction or HLA staining.^6^ Furthermore, human small intestines contain hematopoietic stem and progenitor cells,^17^ and this has been shown to lead to multilineage chimerism.

Conversely, recipient-derived lymphocytes have been shown to replace donor-derived lymphocytes in the gut associated lymphoid tissue of transplanted small intestine.^18,19^ Therefore, cellular rejection and GvHD may depend on the interplay between host and donor-derived lymphocytes in the recipient and in the transplanted organ.

Further to this concept, supportive evidence comes from studies manipulating the balance between lymphocytes derived from the donor relative to those derived from the recipient. The use of rabbit ATG to lymphodeplete the recipient has been shown to reduce cellular rejection, but it does not reduce GvHD risk.^6,20^

Bone marrow transplant patients who are immunodeficient secondary to chemotherapy- or radiotherapy-based conditioning are prone to the development of GvHD. This is potentially because of lack of competition of donor-derived lymphocytes from recipient-derived lymphocytes. Analogous to this, studies of patients with congenital immunodeficiency show that they are more prone to GvHD, again because of lack of competition.^21,22^

Furthermore, studies involving the spleen, the largest lymphoid organ, have alluded to this interplay between donor- and recipient-derived lymphocytes. Removal of the recipient’s spleen is associated with an decreased risk of graft rejection but increased risk of GvHD.^4,23-25^ A study removing the recipient spleen and transplanting the donor spleen together with small intestine found reduced rejection but with slightly higher GvHD.^26^ In our patients undergoing transplantation, spleen-preserving techniques were practiced and allowed for the spleen to be preserved in all but one patient.

Consequently, it has been postulated that GvHD occurs when recipient lymphocytes are outcompeted by donor-derived lymphocytes, whereas cellular rejection may occur when donor-derived lymphocytes are outcompeted by recipient lymphocytes. Variables such as immunosuppression, splenectomy, transplantation of the donor spleen, and volume of intestine transplanted relative to recipient size can all affect this balance.

In our study, the improvement in clinical outcomes after a reduction in immunosuppression may be because of permitting recipient lymphocytes to have a host vs graft effect and outcompete donor-derived lymphocytes. Such a strategy may lead to an increased risk of cellular rejection, but we used steroids and ECP to prevent this, and so, rejection was not seen in our patients.

A limitation of our study is a relatively small number of patients in cohort 2 because of reduction in immunosuppression being a relatively new strategy. Furthermore, we acknowledge the possibility of temporal bias as a further limitation of the study, because of cohort 2 being more recently treated than cohort 1. Nevertheless, the improvement in clinical outcomes and survival with reduction in immunosuppression was profound and statistically significant.
